# Elucidation of the RNA Recognition Code for Pentatricopeptide Repeat Proteins Involved in Organelle RNA Editing in Plants

**DOI:** 10.1371/journal.pone.0057286

**Published:** 2013-03-05

**Authors:** Yusuke Yagi, Shimpei Hayashi, Keiko Kobayashi, Takashi Hirayama, Takahiro Nakamura

**Affiliations:** 1 Faculty of Agriculture, Kyushu University, Fukuoka, Japan; 2 Institute of Advanced Study, Kyushu University, Fukuoka, Japan; 3 Genetically Modified Organism Research Center, National Institute of Agrobiological Sciences, Ibaraki, Japan; 4 Institute of Plant Science and Resources, Okayama University, Okayama, Japan; 5 Biotron Application Center, Kyushu University, Fukuoka, Japan; Colorado State University, United States of America

## Abstract

Pentatricopeptide repeat (PPR) proteins are eukaryotic RNA-binding proteins that are commonly found in plants. Organelle transcript processing and stability are mediated by PPR proteins in a gene-specific manner through recognition by tandem arrays of degenerate 35-amino-acid repeating units, the PPR motifs. However, the sequence-specific RNA recognition mechanism of the PPR protein remains largely unknown. Here, we show the principle underlying RNA recognition for PPR proteins involved in RNA editing. The distance between the PPR-RNA alignment and the editable C was shown to be conserved. Amino acid variation at 3 particular positions within the motif determined recognition of a specific RNA in a programmable manner, with a 1-motif to 1-nucleotide correspondence, with no gap sequence. Data from the decoded nucleotide frequencies for these 3 amino acids were used to assign accurate interacting sites to several PPR proteins for RNA editing and to predict the target site for an uncharacterized PPR protein.

## Introduction

Plant mitochondria and chloroplasts, believed to have been acquired during ancient endosymbiotic events, participate in cellular biogenesis as factories for energy production, photosynthesis, and metabolite synthesis [1], [2]. These organelles contain limited genetic information; as such, numerous nuclear-encoded factors are imported into them to perform various cellular functions. Nuclear-encoded pentatricopeptide repeat (PPR) motif-containing proteins have emerged as important regulators of organelle gene expression [3], [4]. PPR proteins are eukaryote-specific and widely distributed in the plant kingdom, e.g., *Arabidopsis thaliana* contains approximately 450 PPR proteins [5]. As sequence-specific RNA binding proteins, they facilitate various post-transcriptional events, including RNA editing, splicing, cleavage, RNA stability, and translation [3], [4].

RNA editing is the process of altering the sequence of an RNA encoded by the genome. In plants, organellar RNAs are subjected to cytidine (C) to uridine (U), and less frequently U to C, conversion [6]. About 30 RNA editing sites have been found in chloroplast RNA, while more than 400 have been identified in RNA from the mitochondria. To date, roughly 30 individual PPR proteins have been assigned to 1 or several targets each by connecting dysfunctional genes with the loss of RNA editing at specific sites both in chloroplasts and mitochondria [7], [8].

PPR proteins are defined by the presence of degenerate 35-amino-acid units, termed PPR motifs, repeated in tandem up to 30 times [5]. The solved structure of a mitochondrial RNA polymerase containing 2 PPR motifs revealed that the principle configuration of the PPR motif is a pair of antiparallel α-helices, as predicted [9]. Indeed, proteins containing PPR tracts display a helical repeat architecture [10], which is predicted to form a solenoid structure. Transcription activator-like effector (TALE) and pumilio and FBF homology (PUF) repeats are also known to generate helical repeat structures responsible for the interaction of proteins with DNA and RNA, respectively [11], [12]. In these repeats, 1 motif corresponds to 1 base, and the amino acids at particular positions determine the nucleotide-binding specificity. Tandem arrays of PPR motifs within PPR proteins have been assumed to determine the sequence specificity of the protein's RNA-binding activity. Several residues have been suggested as nucleobase-interacting residues [13], [14].

The PPR proteins involved in RNA editing exhibit a characteristic motif organization, which includes a PPR tract followed by additional C-terminal motifs (E, E+, and DYW, although the E+ and DYW motifs are often missing) [15]. A short stretch at the end of the E motif is required for C to U conversion, rather than for recognition of the *cis*-element, which is found at positions -20 to +5 surrounding the editing site [16]. Tandem arrays of PPR motifs within the protein are thought to recognize the upstream nucleotide sequence for the editable C residue [17], [18], [19].

Here, we computationally identified the RNA recognition code for PPR proteins using 24 characterized PPR proteins involved in RNA editing. From this data, we propose the molecular basis for PPR-RNA recognition for RNA editing in plant organelles. Editing PPR proteins indeed recognize the upstream sequence for the editable C residue. The distance between the PPR-RNA alignment and the editable C was shown to be conserved. Furthermore, 1 PPR motif corresponds to 1 nucleotide, and amino acid variation at 3 particular positions confers RNA target specificity in a predictable manner.

Another very recent study demonstrated that PPR tracts bind specific RNA nucleotides via combinatorial action of 2 positions of amino acids in each repeat (6 and 1′; residues 4 and ii, respectively, as used in this study) by computational and biochemical analyses using a well-characterized PPR protein, PPR10, which is involved in RNA stabilization and translation [20]. They showed that the (Thr, Asp) and (Thr, Asn) at the positions (6, 1′) are responsible for binding guanine (G) and adenine (A), respectively. Moreover, (Asn, Asp/Asn/Ser) combinations at the positions (6, 1′) correlate with recognition of pyrimidines, with a significant preference of (Asn, Asp) to U over C. The roles of the 2 amino acids (positions 6 and 1′; residues 4 and ii, respectively, in this study) identified here were very similar to the former observation [20]. We further show the involvement of an additional amino acid (residue 1) in RNA recognition.

The RNA recognition code comprising the 3 amino acids facilitates *in silico* prediction of native RNA targets for several PPR proteins involved in RNA editing, suggesting that the identified RNA recognition code could explain the sequence-specific RNA recognition of PPR proteins.

## Results

### Survey of the amino acids determining nucleotide-specific interactions

**Figure 1:**
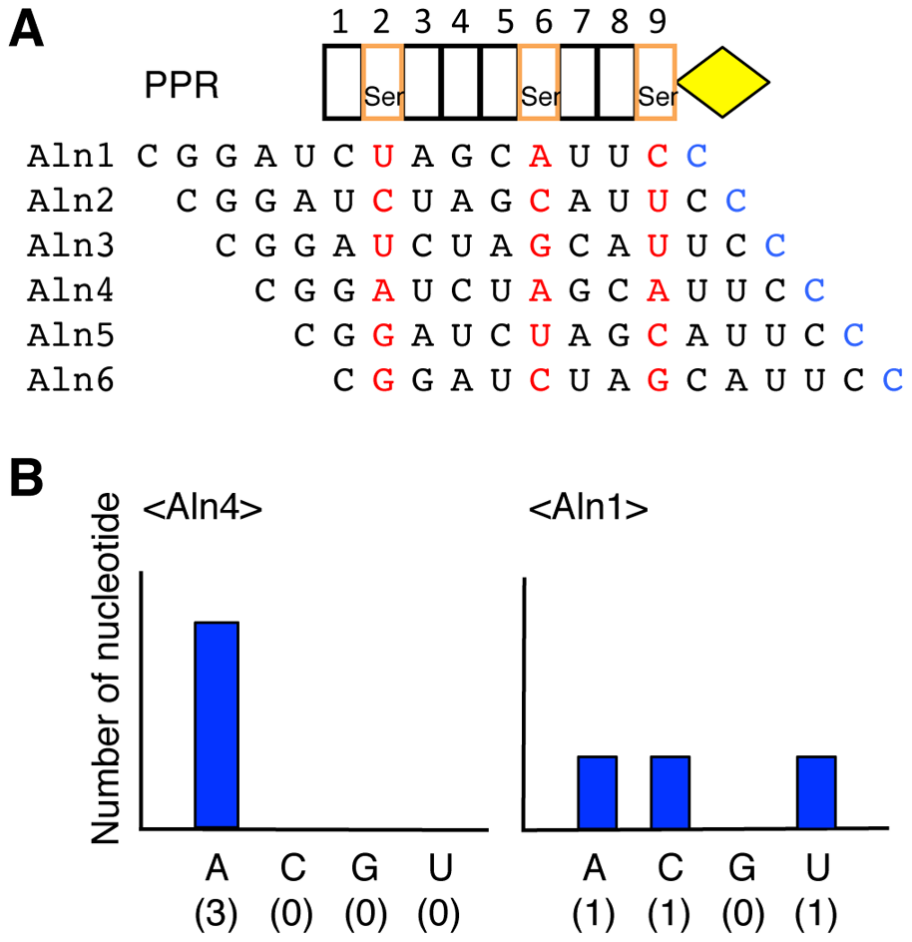
Strategy for screening nucleotide specifying residues (NSRs) for the PPR motif. Potential NSRs were computationally surveyed by estimating the low variability in the association between amino acids in PPR motifs and nucleotides surrounding editing sites. (**A**) The RNA-editing PPR protein contains tandem repeats of the PPR motif (box) and an E motif (diamond) at the C-terminus. The PPR motifs of the editing-type PPR protein were aligned with the corresponding target RNA sequence in various positions, using a 1-motif to 1-nucleotide correspondence, in a contiguous linear manner. Alignment 1 (Aln1) was registered by fitting the last PPR motif of the protein to 1 nucleotide upstream of the editable cytosine residue (shown in blue). The nucleotide sequence was then moved toward the right, 1 nucleotide at a time, for Aln2–6. The association between amino acids in the PPR motif and the corresponding nucleotides was determined for each alignment. (**B**) Expected results for the statistical analysis. If the amino acids at particular positions are responsible for RNA recognition (e.g., Ser is observed at the residue 4 in the second, sixth, and ninth motifs), reduced variability should be observed between the type of amino acid and the specific nucleotide in a particular alignment (*left panel*; Aln4); if not, high variability would be expected (*right panel*; Aln1).

**Figure 2:**
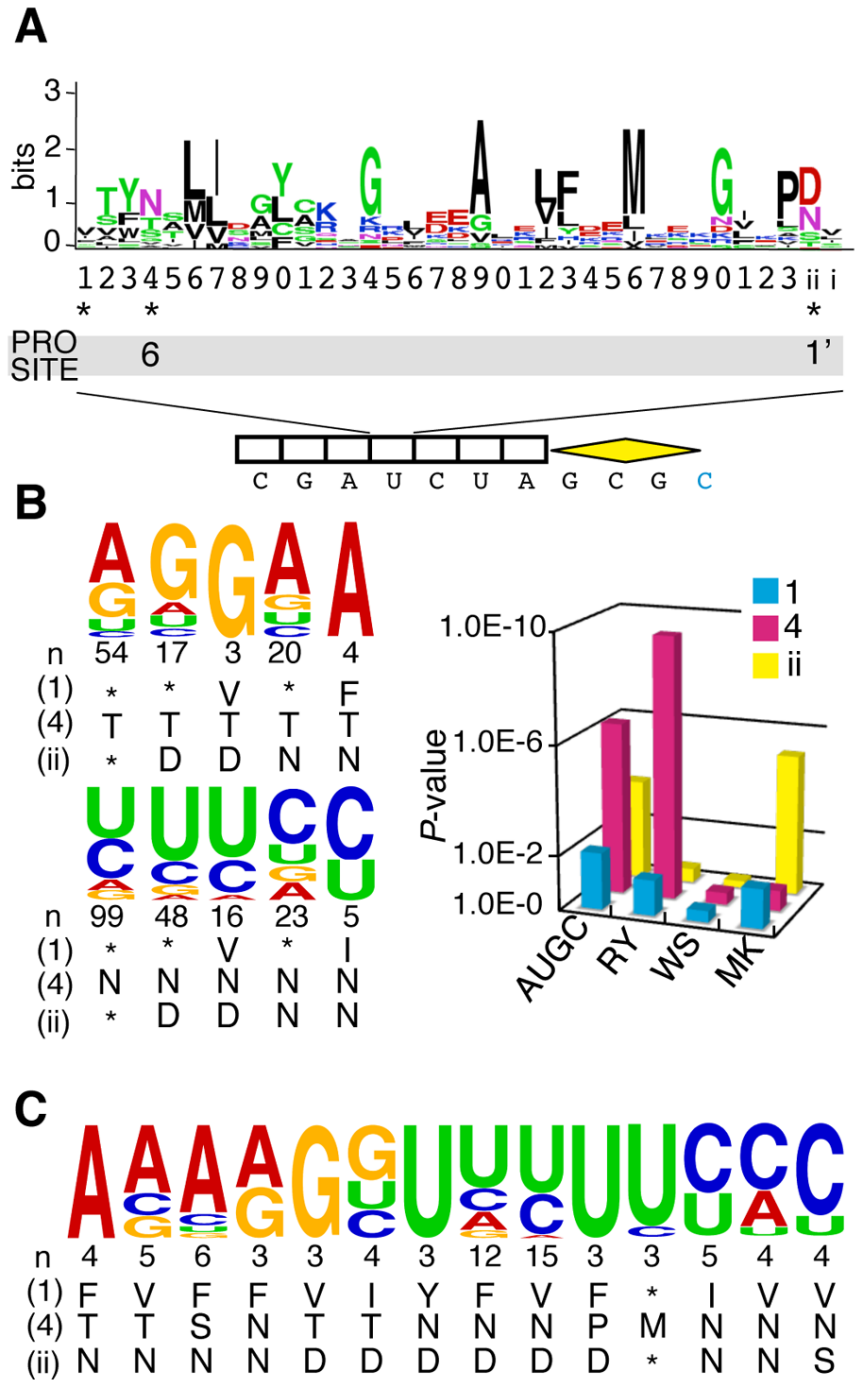
Principles underlying PPR-RNA recognition. (**A**) Consensus amino acids for the PPR motif. The sequence logo was derived from 5668 individual PPR motifs (PROSITE accession no. PS51375). Residues 1, 4, and “ii” (position -2) were determined to be nucleotide-specifying residues (NSRs; asterisks). Previously identified residues for RNA recognition (6 and 1′, [20]) are also shown according to the PROSITE model. The last PPR motif was located 4 nucleotides before the editable C residue (Aln4 in Figure 1). (**B**) NSR-deduced nucleotide frequencies. Nucleotide frequencies, determined according to the NSRs, are displayed in a logo (*left panel*). * indicates any amino acid; “n” indicates the occurrence frequencies of the NSRs in 327 *Arabidopsis* PRR-motifs. The nucleotide-specifying capacity of a residue was deduced from the low variability in the association between the individual amino acids and nucleotides and is presented as a *P*-value (*right panel*). The analysis was conducted for specific nucleotides (i.e., A, U, G, or C), purine/pyrimidine (R or Y), presence or absence of hydrogen bond groups (W or S), and presence or absence of amino/keto (M or K) groups, in alignment 4 (Aln4). (**C**) Example for deduced nucleotide frequencies by various combinations of NSRs.

We here investigated the mechanism that determines PPR-RNA interactions, which may be analogous to that of TALE and PUF repeats [11], [12]. The analysis was conducted informatically by using 24 PPR proteins (containing a total of 327 PPR motifs) characterized as being involved in RNA editing and their target RNA sequences in *Arabidopsis* (Table S1). To test the hypothesis that the PPR tract would recognize the upstream nucleotide sequence for the editable C residue [17], [18], [19], we aligned the PPR motifs and nucleotide sequences upstream of these *cis*-elements in various configurations, with a 1-motif to 1-nucleotide ratio in a linear, contiguous fashion (Figure 1A). Then, we informatically surveyed the position(s) of the nucleotide-specifying residue (NSR), which should display low variability in the association between the type of amino acid and the nucleotide (e.g., Figure 1B). Significantly low variability (*P*<0.01) was observed only for amino acids 1, 4, and “ii” (i.e., -2) in a particular alignment (Aln 4; Figure 2 & S1B) in which the last PPR motif was located 4 nucleotides before the editable C residue. The “ii” indicates the amino acid located 2 residues before the first amino acid of the next PPR motif [14]. The amino acids 4 and “ii” corresponded to the previously identified residues (positions 6 and 1′, respectively, in ref. 20) determining sequence-specific PPR-RNA recognition. This analysis suggested a detailed mechanism for RNA base recognition by the PPR motif (Figure 2B). Residue 4 appeared to be the most important residue for RNA recognition (*P* = 10^−7^), mainly discriminating purine or pyrimidine groups (RY; A&G or U&C). The next most important residue was the “ii” residue (*P* = 10^−4^), which recognized amino or keto groups (MK; A&C or G&U). Finally, residue 1 seemed to be less important (*P* = 10^−3^), but still provided an additional constraint to binding nucleotides. This analysis also suggested the putative RNA recognition code for various combinations of the 3 amino acids in the PPR motifs (Figure 2C).

PPR family proteins are divided into 2 subfamilies, P (classical P) and PLS. Editing-type PPR proteins belong to the PLS subfamily, which contains tandem arrays of the canonical PPR motifs P (35 amino acids), PPR-like L (long, 35 or 36 amino acids), and PPR-like S (short, 31 amino acids). The L-type motifs were suggested to not recognize nucleotide bases in a former study [20] due to distinct amino acid representation at residue 4 in the L motif (position 6 in ref. 20) from that in P and S motifs. Indeed, “Asn” was the most abundant amino acid found at residue 4 in P and S motifs, but was rarely found in the L motif (Figure S2). However, we found that several L-specific amino acids displayed preferences for particular nucleotide bases, e.g., “Pro” at residue 4 was rich only in the L motif and preferred to recognize “U” (FPD, Figure 2C, Figure S2 and Table S3), suggesting the involvement of at least a subset of L motifs in nucleotide base recognition.

### Computational assignment of PPR proteins to their editing sites

**Figure 3:**
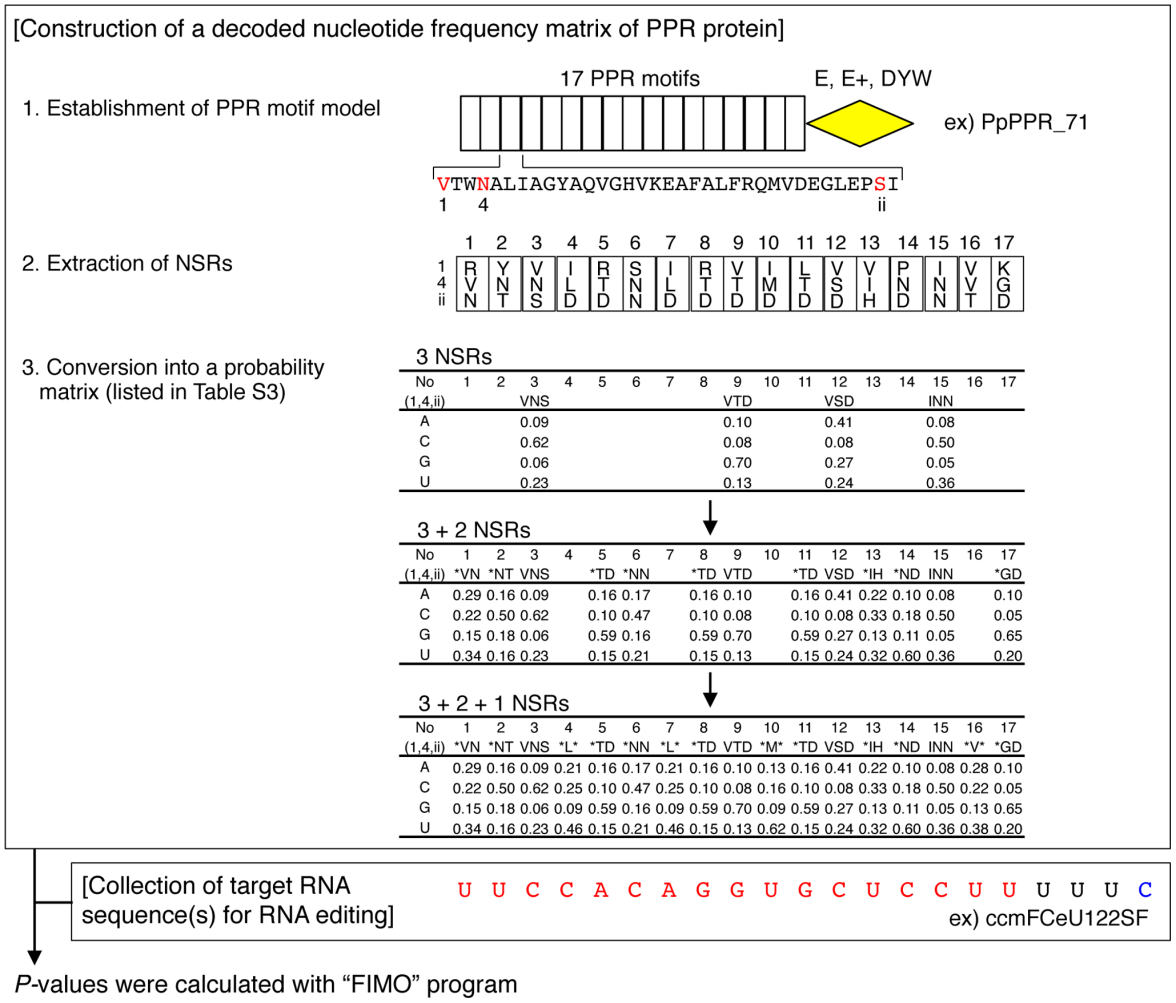
Flowchart for computational target assignment for PPR proteins. The analysis was initiated from the established PPR motif model of a protein of interest, PpPPR_71 for example. Information regarding the nucleotide-specifying residues (NSRs; residues 1, 4, and “ii”) was extracted from the PPR motifs and converted into a probability matrix that indicated the decoded nucleotide frequency for each of the 3 NSRs (residues 1, 4, and ii; listed in Table S3). If a PPR motif did not coincide with all 3 NSRs, it was converted into a matrix for 2 NSRs (residues 4 and ii). The single NSR (residue 4) was used for any remaining PPR motifs. In parallel, target RNA sequences for RNA editing were prepared, corresponding to alignment 4 (Aln4; the sequence upstream from the -4 nucleotide for the editable cytosine residue; highlighted in red). The target sequence for RNA editing (ccmFCeU122SF) by the PpPPR_71 protein is shown as an example. The PPR decoded nucleotide frequency matrix for the protein and nucleotide sequence were analyzed by the FIMO program, which calculated a *P*-value as a measure of the pattern matching score between the PPR protein and the nucleotide sequence.

**Figure 4:**
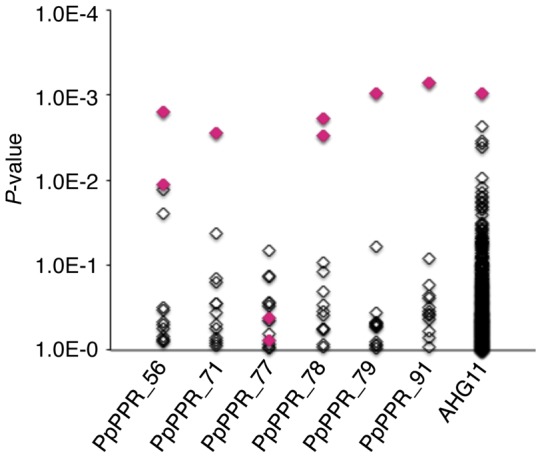
Computational target assignment for PPR proteins. The targets for *Physcomitrella patens* PPR proteins (PpPPR_56, 71, 77, 78, 79, and 91) were computationally assigned against 13 editing sites in *Physcomitrella patens*, using a probability matrix (Table S3) and the FIMO program. Diamonds represent *P*-values indicating a matching score for the respective editing site. Correctly identified editing sites are highlighted in red. The analysis was also performed for an uncharacterized *Arabidopsis* PPR protein, AHG11, against 530 editing sites in *Arabidopsis* mitochondria and chloroplasts.

To verify the role of these 3 amino acids in PPR-RNA recognition, the data from the decoded nucleotide frequencies for the 3 amino acids (Table S3) were used for computational target assignment of characterized PPR proteins in a moss species, *Physcomitrella patens*, which contains 13 editing sites (see detailed procedure in Figure 3) [21], [22]. Currently, 6 PPR proteins (PpPPR_56, PpPPR_71, PpPPR_77, PpPPR_78, PpPPR_79, and PpPPR_91) have been identified as essential for the recognition of 9 editing sites [23], [24], [25], [26]. Our analyses showed that 5 of these 6 characterized moss PPR proteins could be accurately assigned to their editing site(s) using the highest *P*-values obtained (Figure 4), implying that these 3 amino acids are NSRs and contain an RNA-recognition code to explain specific PPR-RNA interactions.

We next expanded our analysis to *Arabidopsis*, which contains more editing sites than the moss plant (34 and 496 sites for chloroplast and mitochondrial genomes, respectively). We first performed a benchmark test using 24 PPR proteins that were included in the initial dataset. Ten out of 13 chloroplast PPR proteins were assigned to at least 1 of their editing sites using the highest *P*-values from the 34 editing sites (Figure S3A). Eight out of 11 mitochondrial proteins were assigned to their editing sites in the top-20 hit list among the 496 mitochondrial editing sites (Figure S3B). Similar results were also obtained from the target assignment tests for 4 recently identified mitochondrial PPR proteins in *Arabidopsis* (MEF3, MEF7, MEF29, and SLG1; Figure S4).

From the results of this benchmark test, we analyzed a gene encoding an uncharacterized PPR protein, abscisic acid hypersensitive germination 11 (AHG11), which was isolated during mutant screening for the abscisic acid signaling pathway. The AHG11 protein exhibits a structure typical of RNA-editing PPR proteins (12 PPRs, E and E+ motifs) [27]. Using our approach, we predicted its target editing site(s) among the 530 *Arabidopsis* editing sites (496 and 34 editing sites in mitochondria and chloroplasts, respectively; Figure 4). The editing status was experimentally verified for more than 400 mitochondrial and all known chloroplast editing sites, with a focus on the top-20 hit list [27]. Our analysis revealed that RNA editing of *nad4_376*, the target exhibiting the highest score, was deficient only in the mutant strain (Figure 4).

Next, we attempted to assign target sites from the whole organelle genome, i.e., a dataset of approximately 10^5^ nucleotides, for several PPR proteins. The genome scan showed that only CRR21 was assigned to the correct target sequence with best matching score (Table 1) in the chloroplast genome using the probability matrix obtained from 24 PPR proteins in *Arabidopsis* (327 PPR motifs; Table S3); the other proteins were not assigned to correct sequences. However, the assignment accuracy could be improved by further refinement of the PPR code via incorporation of additional information from the 137 PPR motifs of the 5 *Physcomitrella patens* PPR proteins and 3 recently characterized *Arabidopsis* PPR proteins (464 PPR motifs; Table S4). In addition, target assignment accuracies declined significantly when the prediction was performed without the 3 letters code (3 NSRs) containing a newly identified NSR of residue 1. This result again indicated the importance of residue 1 for RNA recognition. Taken together, our data demonstrate that elucidation of the PPR code will facilitate the identification of target RNA in plants and non-plant tissues since the trends for amino acid appearance of the 3 NSRs are comparable among plants, humans, and trypanosomes, with the exception of yeast, in which the PPR model has been recently revised (Figure S5).

**Table 1:** Target assignment to the chloroplast genome.

	At code		At + Pp code		At + Pp code (w/o NSR 1)	
Protein	*P*-value	Rank	*P*-value	Rank	*P*-value	Rank
CRR21	9.90E-10	1	4.50E-09	1	5.39E-07	1
CRR4	4.00E-06	8	6.90E-06	7	7.67E-06	8
LPA66	1.30E-05	4	9.48E-06	2	2.28E-04	115
OTP80	9.00E-05	39	2.48E-05	14	1.36E-04	69
OTP81	2.50E-05	28	3.05E-05	43	2.63E-04	390
YS1	4.70E-05	48	3.01E-06	6	2.77E-04	211

Six PPR proteins were used for target assignment against the *Arabidopsis* chloroplast genome (154,478 bp) with the PPR code for 3 NSRs (residues 1, 4, and ii) extracted from 327 PPR motifs in *Arabidopsis* (At; Table S3), or from 464 PPR motifs in *Arabidopsis* and *Physcomitrella patens* (At + Pp; Table S4). The assignment was also performed with the PPR code excluding residue 1 (only residues 4 and ii; 2 NSRs and 1 NSR of “At + Pp” code).

## Discussion

In this study, we showed that 3 amino acids (1, 4, and “ii”[-2]) comprised the NSRs of PPR motifs, determining the sequence-specific recognition of target RNA sequences. Moreover, we showed that the decoded nucleotide frequency for the 3 NSRs could facilitate *in silico* prediction of RNA targets for editing PPR proteins. This observation further strengthened the involvement of residues 4 and “ii” in sequence-specific PPR-RNA recognition; these residues were identified as NSRs in another *in silico* analysis using different PPR molecules (positions 6 and 1′ in ref. 20). The present study further identified residue 1 (position 3 in ref.20) as an additional NSR as well as various RNA recognition codes for the degenerate 3 NSRs. Although residue 1 seems to be less important than other NSRs (4, ii; Figure 2), its significance was shown by improvement of target assignment accuracy against the whole chloroplast genome sequence (Table 1). Barkan et al [20] suggested the involvement of position 4′ (corresponding to residue 2 of the next motif in our numbering) in RNA recognition from their analysis of PPR10. However, low variability, indicating sequence-specific RNA recognition, was not observed for this residue (Figure S1B; amino acid 2 in Aln5) in the current study using 24 editing PPR proteins. These observations suggest that the RNA recognition event may involve abnormal use of amino acids at different positions. This possibility should be examined in future studies.

Our study also indicated that the last PPR motif of the editing PPR protein would be located 4 nucleotides before the editable C residue. The L motif, as well as P and S motifs, would be involved in RNA recognition. Moreover, we demonstrated that the PPR tract recognized the upstream nucleotide sequence by a 1-motif to 1-nucleotide correspondence, with no gap sequence, in contrast to the classical P-type PPR protein involved in processes other than RNA editing, e.g., PPR10, CRP1, and HCF152, which contain gaps in the PPR/RNA duplex [20].

This “gap-model” for classical P-type PPR proteins makes computational target prediction difficult due to the large expansion of total possibilities for motif-to-base alignments. The RNA recognition code here could be also applied for classical P-type PPR proteins because representation of NSR was highly correlated in classical P motifs versus P and S motifs in the editing-PPR protein (Figure S2). In addition, the principles of a recently identified RNA recognition code using the classical P-type PPR protein PPR10 [20] were almost identical to the code identified in this study. This fact further supports the generality of the RNA recognition code underlying PLS- and classical P-type PPR proteins. Further analysis will be required for elucidation of the filtering determinants for binding or gap region in order to facilitate accurate target prediction for classical P-type PPR proteins.

Our data demonstrated that the 3 NSRs (1, 4, and “ii”) were located in spatially closed positions in the determined PPR structure (Figure S6A). Biochemical and bioinformatics studies have suggested that these 3 amino acids may be involved in nucleobase interactions [13], [14]. Our previous biochemical studies identified 5 amino acids (1, 4, 8, 12, and “ii”) as RNA-interacting residues. However, low variability, indicating sequence-specific RNA recognition, was not observed for residues 8 or 12 in the statistical survey (Figure S1B). Because residues 8 and 12 are often basic, they may facilitate a general preference for particular RNA molecules. Residue 4 seemed to be the most important residue for PPR-RNA recognition and acted in purine/pyrimidine recognition. This is consistent with a previous study suggesting that editing PPR proteins can distinguish pyrimidines from purines, and, at some positions, can even recognize specific bases [28].

Notably, the NSRs identified in this study were highly diverse, in spite of recognizing only 4 types of ribonucleotides. In addition, variations and locations of code-generating residues for the PPR motif differed from those of other helical repeat proteins containing TALEs and PUFs (Figure S6) [11], [12]. In the future, determination of the structure of PPR-RNA complexes will be necessary to provide further understanding of the fundamental principles of protein-RNA interactions and may allow for the engineering of custom PPR proteins.

The *cis*-element for the recognition of the editing site has been shown to consist of the sequence spanning from 20 nucleotides upstream to 5 nucleotides downstream of the editable C residue [15]. The editing PPR protein contains an additional C-terminal motif (E, E+, and DYW), and the importance of the E motif has been demonstrated [16]. The E motif is predicted to contain 4 repetitions of α helices, corresponding to those of 2 PPR motifs. The additional motif(s), as well as an as-of-yet undefined editing enzyme, may be involved in the recognition of nucleotides in the vicinity of the editable C residues (-3 to +5). It is also possible that the recently identified novel protein family of multiple organellar RNA editing factor (MORF, also known as RNA-editing factor interacting protein [RIP]) is involved in sequence recognition for RNA editing [29], [30]. This may also explain our imperfect computational assignments for the target RNAs (Figure S3). The molecular mechanisms of these processes should be examined in detail in later studies, including further elucidation and refinement of the RNA recognition code for the PPR motif.

Recent analyses have indicated that PPR proteins play a significant role in nuclear–cytoplasmic interactions, including cellular homeostasis and hybrid sterility, in various organisms [31], [32]. Plant PPR proteins have been suggested to be non-redundant and are often involved in cell viability [5]. Notably, the genes encoding PPR proteins constitute 1/50 of the total protein-coding genes present in many terrestrial plants. Thus, the elucidation of the RNA recognition code for PPR protein and *in silico* prediction of their target RNA(s) will facilitate the analysis of PPR proteins and the characteristics of the PPR protein family, and this will hopefully provide a better understanding of the mechanisms that underlie the nuclear control of organelle gene expression, especially in plants.

## Materials and Methods

### Selection of editing factors and their target sequences

Sequences of PPR motifs within *Arabidopsis* PPR proteins were retrieved from the UniProt database (http://www.uniprot.org/) because UniProt provides detailed definitions for degenerated PPR motifs. For *Physcomitrella* PPR proteins, PPR motifs were defined using the PROSITE program (http://prosite.expasy.org/), and their degenerated PPR motifs were estimated from a predicted secondary structure (PSI-Pred; http://bioinf.cs.ucl.ac.uk/psipred/). All examined editing PPR proteins in this study possess contiguous PPR arrays. The amino acid positions were defined as shown in Figure 2 [14]. Residues 1, 4, and “ii” of the PPR motifs of all PPR proteins used in this study, as well as their target sites, are listed in Supplementary Table S1 and S2.

### Statistical survey of the amino acids determining nucleotide specificity

Statistical analysis was performed using 24 editing-type PPR proteins, which contained a combined total of 327 PPR motifs, and their 34 RNA target sequences from *Arabidopsis* (Table S1). The protein and nucleotide sequences were aligned using a 1-motif to 1-nucleotide correspondence in a linear contiguous manner (Figure 1). Alignment 1 (Aln1) was registered by fitting the last PPR motif of the protein to 1 nucleotide upstream of the editable C residue. Similarly, Aln2, Aln3, Aln4, Aln5, and Aln6 were generated by successively sliding the nucleotide sequence toward the right, 1 nucleotide at a time, from the position in Aln1. The nucleotide occurrence frequency was determined to be 1 for a PPR protein involved in a single editing site. For PPR proteins involved in 2 and 3 editing sites, the nucleotide occurrence frequency was calculated as 0.50 and 0.33 points, respectively, if the corresponding nucleotides were diverse. Then, the set of PPR motifs and nucleotides was sorted according to the type of amino acid for each alignment. Low variability between the amino acids and the corresponding nucleotide occurrence frequency was calculated with a chi-square test, using the observed and expected (mean) frequencies (e.g., Figure S1A). The expected frequency was obtained from the total occurrence frequency of the particular nucleotides. The calculation was performed for all amino acid positions within a PPR motif for the above-mentioned 6 alignments (Figure S1B). Frequently used NSRs (≥3) and their nucleotide occurrence frequencies in Aln4 are listed in Table S3 and were used for computational target assignment. The sequence logos in Figure 2 were depicted using WebLogo (http://weblogo.threeplusone.com/).

### Computational target assignment for PPR proteins

Before target assignment, a probability matrix was obtained from the observed base counts (B), with pseudocount correction, to reflect the reliability of the total number of occurrences (N) of respective NSRs, as previously described [33]. The square root of N was used as the pseudocount. The formula is represented as follows:
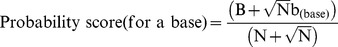
where b_(base)_ indicates the background frequency of nucleotide occurrence.

The procedure for computational target assignment is shown in a flowchart in Figure 3. Briefly, the PPR-motif model for each protein was obtained as described above. Then, the amino acids for an NSR (residues 1, 4, and “ii”) were extracted from all PPR motifs in the protein. The respective PPR motif was converted to a corresponding probability matrix for the 3 NSRs (residues 1, 4, and “ii”), if the corresponding code is listed in Table S3. Any PPR motif that did not coincide with the 3 NSRs was subsequently converted to a probability matrix for 2 NSRs (residues 4 and “ii”). Lastly, the score for a single NSR (residue 4) was used for the remaining PPR motifs. The decoded nucleotide frequency matrix for the protein was subjected to FIMO program in the MEME suite (http://meme.nbcr.net/meme4_6_1/fimo-intro.html), using a nucleotide dataset of sequences upstream of editing sites according to the RNA editing database (http://biologia.unical.it/py_script/overview.html) or the *Arabidopsis* chloroplast genome sequence (AP000423).

### Collection of PPR protein sequences from various organisms

The amino acid sequences of PPR proteins in *Arabidopsis* (*Arabidopsis thaliana*), humans (*Homo sapiens*), and trypanosomes (*Trypanosoma brucei*) were retrieved from the UniProt database. The PPR-motif model for each protein was determined as described above. The sequences and motif architectures for PPR proteins in yeast (*Saccharomyces cerevisiae*) were derived as previously described [34]. Pearson correlation coefficients were estimated for the trend of amino acid variations at the NSR (residues 1, 4, or “ii”) between *Arabidopsis* and non-plant tissues (Figure S5B).

## Supporting Information

Figure S1 **Statistical survey for the nucleotide-specifying residues (NSRs) in a PPR motif**. (**A**) Example of the estimation of low variability between the amino acid and nucleotide. The observed frequency indicates the frequency of actual occurrence of nucleotides at particular amino acids at residue 4 in Aln4. The expected frequency was obtained from the total occurrence frequency of the nucleotides. Low variability was represented by a *P*-value, which was obtained by a chi-square test from the observed and expected frequencies. (**B**) Low variability between the amino acid and the nucleotide was calculated for all positions of amino acids in Aln1–6. Amino acid positions with significantly low randomness (*P*<0.01) are highlighted in red. The blue line indicates a *P*-value of 0.01.
(PDF)

Figure S2 **Variations in the nucleotide-specifying residues (NSRs) in** ***Arabidopsis*** **PPR subtypes.** Occurrence frequencies for residues 1, 4, and ii were estimated for P, L, and S motifs in PLS subfamily proteins and for P motifs in classical P-type PPR subfamily proteins.
(PDF)

Figure S3 **Benchmark test for computational target assignment using characterized PPR proteins in** ***Arabidopsis*** **.** (**A**) *P*-values for previously characterized chloroplast PPR proteins against all 34 chloroplast-editing sites. The diamond represents the *P*-value for the matching score against the editing site. The correct editing site is highlighted in red. (**B**) *P*-values for characterized mitochondrial PPR proteins against all 496 mitochondrial editing sites.
(PDF)

Figure S4 **Computational target assignment for recently identified mitochondrial PPR proteins.** The targets for *Arabidopsis* PPR proteins (MEF3, MEF7, MEF29, and SLG1) were computationally assigned against 496 editing sites in *Arabidopsis* mitochondria, using a probability matrix (Table S3) and the FIMO program.
(PDF)

Figure S5 **Variation of the nucleotide-specifying residues (NSRs) in various organisms.** (**A**) Occurrence frequencies for specific amino acids at residues 1, 4, or ii in PPR motifs from At (*Arabidopsis thaliana*), Hs (*Homo sapiens*), Tb (*Trypanosoma brucei*), and Sc (*Saccharomyces cerevisiae*). The *Arabidopsis* PPR motif contains all PPR subtypes of the P, L, and S motifs. (**B**) Correlation coefficients for NSR variations between plant and nonplant tissues. Pearson correlation coefficients were estimated for trends in amino acid variations at the NSRs (residues 1, 4, or ii) between *Arabidopsis* and nonplant tissues.
(PDF)

Figure S6 **Structures of PPR, PUF, and TALE repeats.** The residue that determines the RNA-binding specificity is shown as a red stick within the front motif. (A) Structures of 2 PPR motifs (residues 263–330; PDB: 3SPA). RNA recognition was determined by residues 1, 4, and “ii” (position -2), shown in red, among the 35 amino acids of the PPR motif. (B) Structures of 2 PUF repeats (residues 996–1067; PDB: 1M8W). RNA recognition was determined by residues 12 and 16 (red) among the 36 amino acids of the PUF repeat. The residue 13 (pink) facilitated RNA binding by stacking the base with the same residue of an adjoining repeat. (C) Structure of 2 TALE repeats (residues 624–691; PDB: 3UGM). DNA recognition was determined by residues 12 and 13 (red) among the 34 amino acids of the TALE repeat.
(PDF)

Table S1 **List of PPR proteins and their target sites.**
(PDF)

Table S2 **List of** ***Physcomitrella patens*** **PPR proteins and newly characterized** ***Arabidopsis*** **PPR proteins, and their target sites.**
(PDF)

Table S3 **List of scoring matrix for NSRs extracted from 327 PPR motifs in 24** ***Arabidopsis*** **PPR proteins.**
(XLS)

Table S4 **List of scoring matrix for NSRs extracted from 464 PPR motifs in 27** ***Arabidopsis*** **and 5** ***Physcomitrella*** **PPR proteins.**
(XLS)
