# Supplementary material for: Elucidation of the RNA Recognition Code for Pentatricopeptide Repeat Proteins Involved in Organelle RNA Editing in Plants
Source: PLoS One. 2013 Mar 5;8(3):e57286. doi: 10.1371/journal.pone.0057286 (PMC3589468; doi:10.1371/journal.pone.0057286)

Figure S1

A

Observed frequency		A	C	D	F	G	H	I	K	L	M	N	P	Q	R	S	T	V	Total
	A	4.3	4.0	0.0	0.0	8.0	0.7	1.8	0.5	2.3	0.0	10.7	1.0	0.0	1.0	15.8	26.7	9.5	86.3
	C	3.2	0.3	0.0	0.0	2.2	0.0	4.5	0.5	4.3	0.3	35.3	0.5	0.0	0.0	5.0	4.0	7.5	67.6
	G	3.0	1.3	1.0	0.0	8.0	0.0	0.8	1.0	0.0	0.0	11.3	1.7	0.0	0.0	8.8	16.2	2.8	56.0
	U	5.5	0.3	0.0	1.0	6.8	0.3	2.8	0.0	8.3	2.7	41.7	13.8	1.0	0.0	12.3	7.2	13.2	117.0
	Total	16.0	6.0	1.0	1.0	25.0	1.0	10.0	2.0	15.0	3.0	99.0	17.0	1.0	1.0	42.0	54.0	33.0	327

Expected frequency		A	C	D	F	G	H	I	K	L	M	N	P	Q	R	S	T	V	Total
	A	4.2	1.6	0.3	0.3	6.6	0.3	2.6	0.5	4.0	0.8	26.1	4.5	0.3	0.3	11.1	14.3	8.7	86.3
	C	3.3	1.2	0.2	0.2	5.2	0.2	2.1	0.4	3.1	0.6	20.5	3.5	0.2	0.2	8.7	11.2	6.8	67.6
	G	2.7	1.0	0.2	0.2	4.3	0.2	1.7	0.3	2.6	0.5	16.9	2.9	0.2	0.2	7.2	9.2	5.6	56.0
	U	5.7	2.1	0.4	0.4	8.9	0.4	3.6	0.7	5.4	1.1	35.4	6.1	0.4	0.4	15.0	19.3	11.8	117.0
	Total	16.0	6.0	1.0	1.0	25.0	1.0	10.0	2.0	15.0	3.0	99.0	17.0	1.0	1.0	42.0	54.0	33.0	327

P-value(Aln4, residue 4) = 5.7×10^{-7}

B

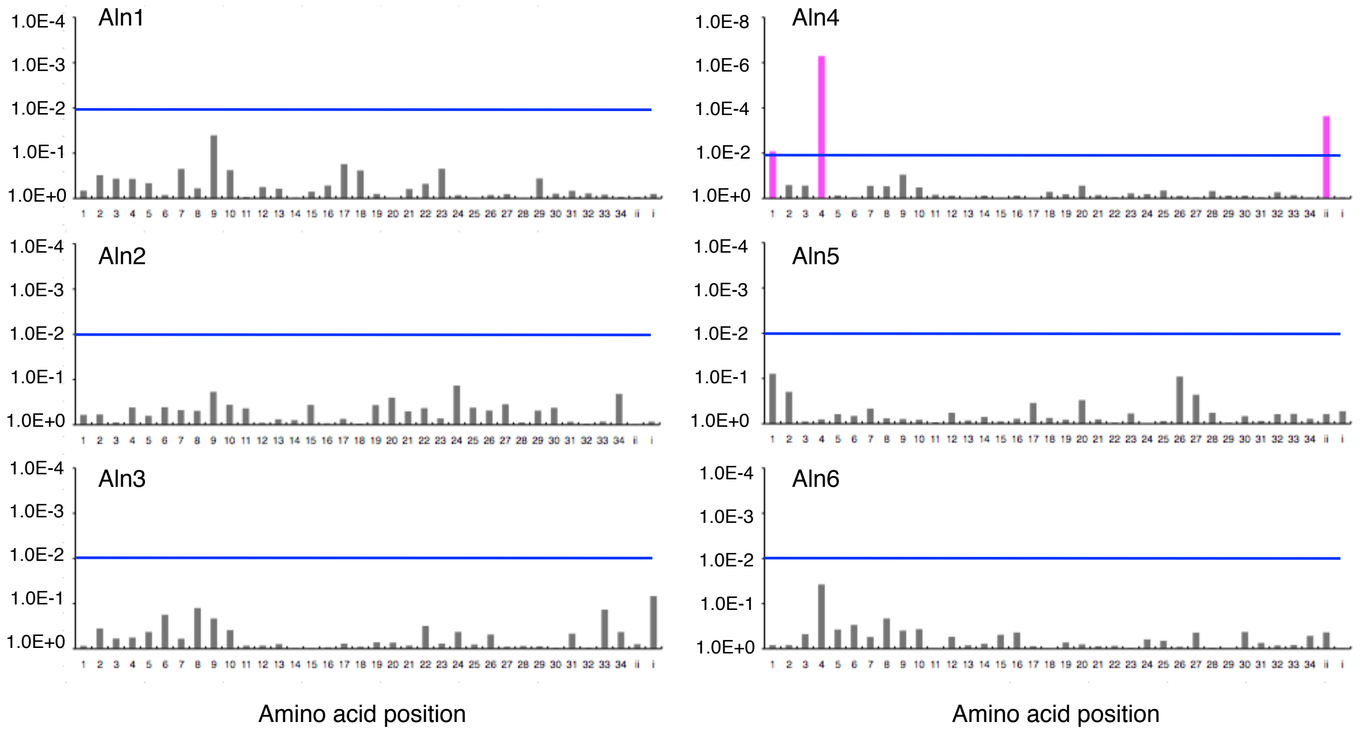

Supplement: Figure S1 — Statistical survey for the nucleotide-specifying residues (NSRs) in a PPR motif. (A) Example of the estimation of low variability between the amino acid and nucleotide. The observed frequency indicates the frequency of actual occurrence of nucleotides at particular amino acids at residue 4 in Aln4. The expected frequency was obtained from the total occurrence frequency of the nucleotides. Low variability was represented by a P-value, which was obtained by a chi-square test from the observed and expected frequencies. (B) Low variability between the amino acid and the nucleotide was calculated for all positions of amino acids in Aln1–6. Amino acid positions with significantly low randomness (P<0.01) are highlighted in red. The blue line indicates a P-value of 0.01. (PDF) [file pone.0057286.s001.pdf]
