# Supplementary figures and images for: Elucidation of the RNA Recognition Code for Pentatricopeptide Repeat Proteins Involved in Organelle RNA Editing in Plants
Source: PLoS One. 2013 Mar 5;8(3):e57286. doi: 10.1371/journal.pone.0057286 (PMC3589468; doi:10.1371/journal.pone.0057286)

### Figure S2

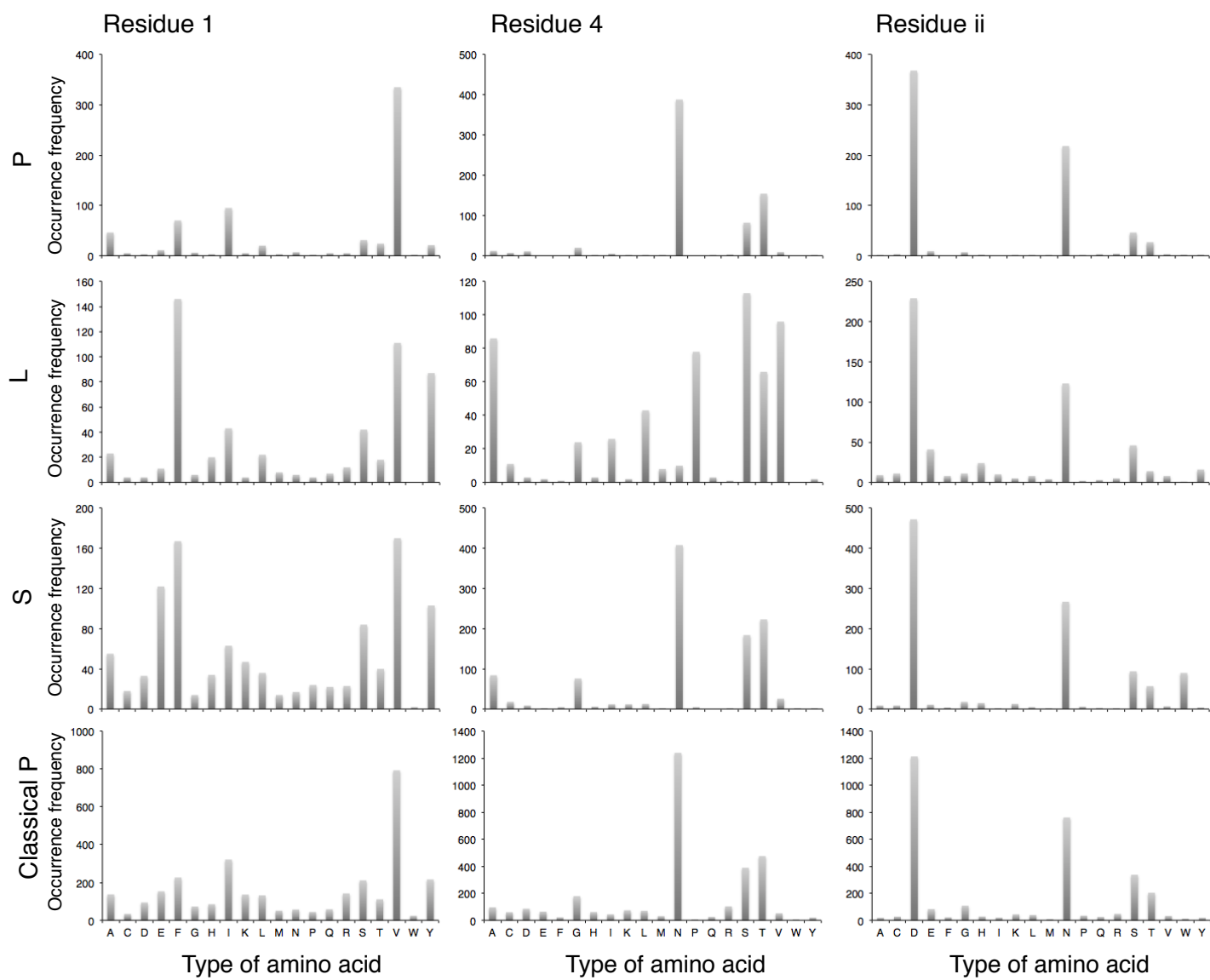

Supplement: Figure S2 — Variations in the nucleotide-specifying residues (NSRs) in Arabidopsis PPR subtypes. Occurrence frequencies for residues 1, 4, and ii were estimated for P, L, and S motifs in PLS subfamily proteins and for P motifs in classical P-type PPR subfamily proteins. (PDF) [file pone.0057286.s002.pdf]

Figure S3

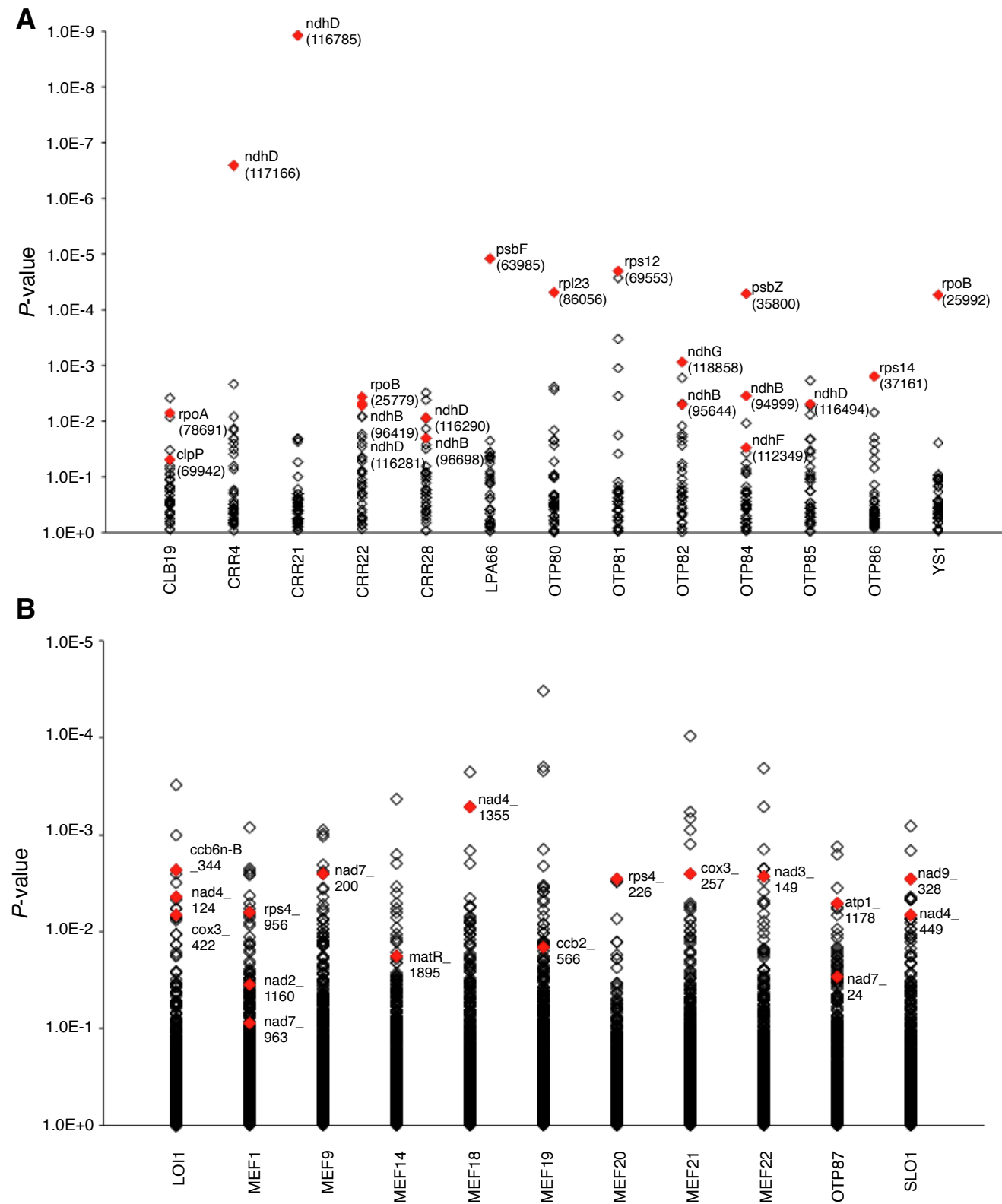

Supplement: Figure S3 — Benchmark test for computational target assignment using characterized PPR proteins in Arabidopsis . (A) P-values for previously characterized chloroplast PPR proteins against all 34 chloroplast-editing sites. The diamond represents the P-value for the matching score against the editing site. The correct editing site is highlighted in red. (B) P-values for characterized mitochondrial PPR proteins against all 496 mitochondrial editing sites. (PDF) [file pone.0057286.s003.pdf]

Figure S4

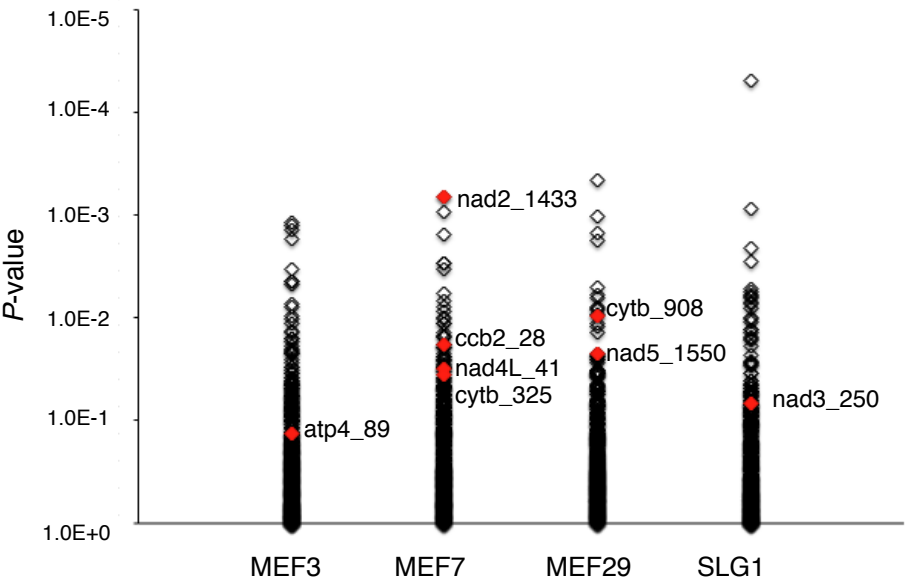

Supplement: Figure S4 — Computational target assignment for recently identified mitochondrial PPR proteins. The targets for Arabidopsis PPR proteins (MEF3, MEF7, MEF29, and SLG1) were computationally assigned against 496 editing sites in Arabidopsis mitochondria, using a probability matrix (Table S3) and the FIMO program. (PDF) [file pone.0057286.s004.pdf]

Figure S5

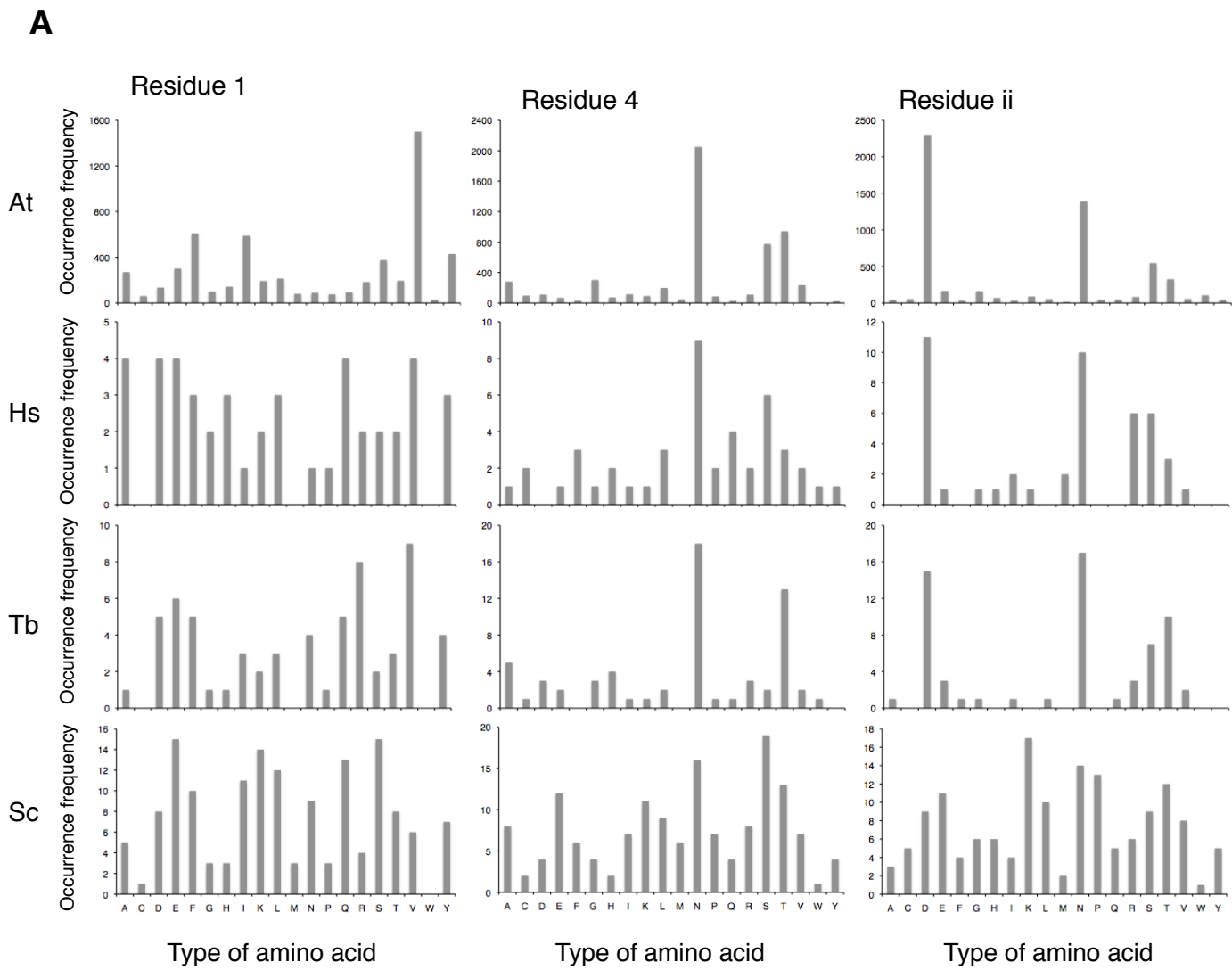

**B**

Correlation coefficient for the NSR variation

|          | 1    | 4    | ii   |
|----------|------|------|------|
| At vs Hs | 0.39 | 0.82 | 0.88 |
| Tb       | 0.61 | 0.91 | 0.88 |
| Sc       | 0.18 | 0.68 | 0.31 |

Supplement: Figure S5 — Variation of the nucleotide-specifying residues (NSRs) in various organisms. (A) Occurrence frequencies for specific amino acids at residues 1, 4, or ii in PPR motifs from At (Arabidopsis thaliana), Hs (Homo sapiens), Tb (Trypanosoma brucei), and Sc (Saccharomyces cerevisiae). The Arabidopsis PPR motif contains all PPR subtypes of the P, L, and S motifs. (B) Correlation coefficients for NSR variations between plant and nonplant tissues. Pearson correlation coefficients were estimated for trends in amino acid variations at the NSRs (residues 1, 4, or ii) between Arabidopsis and nonplant tissues. (PDF) [file pone.0057286.s005.pdf]

**Figure S6**

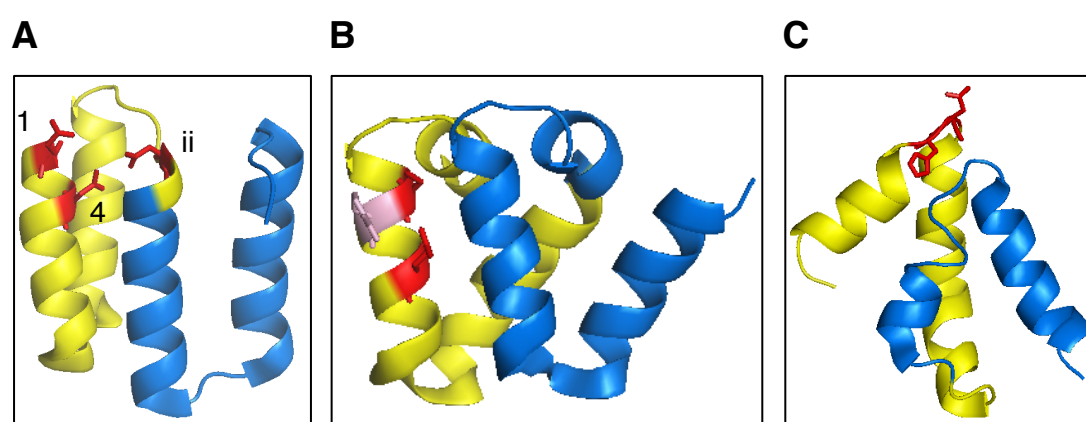

Supplement: Figure S6 — Structures of PPR, PUF, and TALE repeats. The residue that determines the RNA-binding specificity is shown as a red stick within the front motif. (A) Structures of 2 PPR motifs (residues 263–330; PDB: 3SPA). RNA recognition was determined by residues 1, 4, and “ii” (position -2), shown in red, among the 35 amino acids of the PPR motif. (B) Structures of 2 PUF repeats (residues 996–1067; PDB: 1M8W). RNA recognition was determined by residues 12 and 16 (red) among the 36 amino acids of the PUF repeat. The residue 13 (pink) facilitated RNA binding by stacking the base with the same residue of an adjoining repeat. (C) Structure of 2 TALE repeats (residues 624–691; PDB: 3UGM). DNA recognition was determined by residues 12 and 13 (red) among the 34 amino acids of the TALE repeat. (PDF) [file pone.0057286.s006.pdf]
