# Supplementary material for: Elucidation of the RNA Recognition Code for Pentatricopeptide Repeat Proteins Involved in Organelle RNA Editing in Plants
Source: PLoS One. 2013 Mar 5;8(3):e57286. doi: 10.1371/journal.pone.0057286 (PMC3589468; doi:10.1371/journal.pone.0057286)
Supplement: Table S1 — List of PPR proteins and their target sites. (PDF) [file pone.0057286.s007.pdf]

Table S1. List of PPR proteins and their target sites

Name	Species*	AGI number	Localization	NSRs in the motifs	Target RNA sequence	Editing site	(ref)
CLB19	At	At1g05750	Chl	VTN-IIH-MTN-VNW-AND-VIN-RNT-VND-VTR-EGI	acacgugcaa<br>agaagcccaa	rpoA(78691)<br>clpP(69942)	[35]
CRR21	At	At5g55740	Chl	EGN-YTN-FAD-FPC-FSN-VNT-VSD-ITD-VND-VAD-VSD-INN-INN-ITN-FTL-STE-PND-ITC-EGD	auguacagcggucaaauag	ndhD(116785)	[16]
CRR22	At	At1g11290	Chl	FTL-VHV-YTD-FTD-VNS-IVL-NTN-VNT-VMN-SNT-VND-FVN-FTH-TNN-VLS-DGA	aagcuuucccuagccc<br>uaugcagcuucaacau<br>auaucuauucuaguuc	ndhB(96419)<br>ndhD(116281)<br>rpoB(25779)	[36]
CRR28	At	At1g59720	Chl	FGS-FND-HPD-YNS-VND-YQD-LND-ANN-VVA-EGD	aucuuuguag<br>auuuaugcag	ndhB(96698)<br>ndhD(116290)	[36]
CRR4	At	At2g45350	Chl	FND-FSD-FND-VNN-IND-INW-TDD-VND-TVG-KVS-DND-IVR-QGN	uaucuugucuuua	ndhD(117166)	[37]
LOI1	At	At4g14850	Mt	DGP-FNN-VTN-FPD-FCN-ENN-ICD-SNW-SAS-FST-FSN-VNN-MVG-EST	ugauacgauuaauu<br>uaauacccuuauuc<br>uaauauugugacuu	nad4(161816)<br>cox3(218701)<br>ccb203(257133)	[38]
LPA66	At	At5g48910	Chl	LAN-FNN-FPD-FSE-VNS-VNN-VVD-VSN-ISS-VIR-EGD	uguaccuaccg	psbF(63985)	[39]
MEF1	At	At5g52630	Mt	NCI-LNS-TSD-HPD-FSN-VSN-YSS-FSN-GNN-ILT-KAW	accuacucauu<br>acagaagguuu<br>aacgaauccua	rps4(82161)<br>nad7(137931)<br>nad2(329886)	[40]
MEF9	At	At1g62260	Mt	VNW-TSD-FNW-ATA-VLL-YNN-VND-INW-MSH-VND-HTD-PNE-INS-IVQ-ESD	uucaagcuuuaccuu	nad7(133233)	[41]
MEF14	At	At3g26780	Mt	FNT-SPD-FSI-SSD-MVG-SND-VNN-ISD-ITN-RTN-IVG-EGG	aguacuccaaagcu	matR(144418)	[42]
MEF18	At	At5g19020	Mt	RVN-YND-ANS-VTN-VAR-FTN-VND-VGS-VVY-FAH-AND-FND-IVN-NAT-SNN-IVD-KGD	uccauaaauucuccg	nad4(167599)	[43]
MEF19	At	At3g05240	Mt	ISS-YND-FPN-YTN-VGN-TVN-ITT-VND-VLD-ACD-ITD-ILT-EGN	uuauugauugguu	ccb206(239125)	[43]
MEF20	At	At3g18970	Mt	RVS-LTW-AGT-TVD-FTN-FTN-ITV-EGD	cacgaaag	rps4(82891)	[43]
MEF21	At	At2g20540	Mt	FTN-FND-FPH-VND-INW-ASD-IIQ-GND-ISN-ILK-EGD	acuucgauaug	cox3(218536)	[43]
MEF22	At	At3g12770	Mt	FTQ-FND-FPD-FNT-VTD-VVE-DIN-IND-ITD-FSD-VSN-VLQ-QAG	ugugguuucgauc	nad3(260858)	[43]
otp80	At	At5g59200	Chl	FFN-YTN-VSS-SLD-VTD-VTN-FVS-FND-INN-VVQ-EGD	ugucgaaucag	rpl23(86055)	[28]
otp81	At	At2g29760	Chl	FNN-YPD-FND-VNS-VVN-TND-VTW-ASN-IVN-HSD-FSN-VTE-KAS	auucuuuuugaaa	rps12(69553)	[28]
otp82	At	At1g08070	Chl	LNN-YPD-YTY-AKD-VND-SVN-KND-INN-VLA-STS-SND-IVK-EGD	uuaguuuuucugg<br>guagcugcuucag	ndhG(118858)<br>ndhB(95644)	[28]
otp84	At	At3g57430	Chl	EID-YPS-TNN-VNS-FVN-FND-VND-FSN-FSW-AAN-TAD-FNW-TTN-IMD-ASN-INN-VIS-DAN	auccacuuacuucuauua<br>uugcauuaauuauuacuu<br>gugugauagcaucuacua	ndhF(112349)<br>psbZ(35800)<br>ndhB(94999)	[28]
otp85	At	At2g02980	Chl	VND-YND-YPC-VNN-ILY-KTD-QSD-ILS-KGL	uugccguca	ndhD(116494)	[28]
otp86	At	At3g63370	Chl	FGT-FNG-SPT-FND-VNN-YVE-YND-VND-VTN-QND-ITD-MGD-VND-VTD-VLE-SVG-LTD-ILW-EVT	uaucauuugauucgucgau	rps14(37161)	[28]
otp87	At	At1g74600	Mt	FKD-VNN-IGY-VSN-YND-YSD-FTS-VTN-CTD-SAR-NND-FCD-TSD-AAD-SAG-DSD-VSD-FSE-SSD-ITD-VVE-RVW	ccaugacgacuaggaaaaggca<br>gcuaugaaacaaguaugcggua	nad7(132094)<br>atp1(82180)	[44]
SLO1	At	At2g22410	Mt	FND-FPV-HND-VND-VIT-PNT-VTW-AGD-IIN-ATN-LTD-IIQ-KSD	uuucccgaaagcg<br>auuuccaucagcc	nad4(162141)<br>nad9(23908)	[45]
YS1	At	At3g22690	Chl	FND-YPD-FNW-SCN-VVN-LSN-DND-ILW-NNT-VNW-TSD-VMD-RTD-STD-VVE-VGN	augaauucccuuggaa	rpoB(25779)	[46]

*At, Arabidopsis thaliana

References

16. Okuda K, Myouga F, Motohashi R, Shinozaki K, Shikanai T (2007) Conserved domain structure of pentatricopeptide repeat proteins involved in chloroplast RNA editing. Proc Natl Acad Sci USA 104: 8178-8183.
28. Hammani K, Okuda K, Tanz SK, Chateigner-Boutin AL, Shikanai T, et al. (2009) A study of new Arabidopsis chloroplast RNA editing mutants reveals general features of editing factors and their target sites. Plant Cell 21: 3686-3699.
35. Chateigner-Boutin A-L, Ramos-Vega M, Guevara-García A, Andrés C, de la Luz Gutiérrez-Nava Ma, et al. (2008) CLB19, a pentatricopeptide repeat protein required for editing of rpoA and clpP chloroplast transcripts. Plant J 56: 590-602.
36. Okuda K, Chateigner-Boutin AL, Nakamura T, Delannoy E, Sugita M, et al. (2009) Pentatricopeptide repeat proteins with the DYW motif have distinct molecular functions in RNA editing and RNA cleavage in Arabidopsis chloroplasts. Plant Cell 21: 146-156.
37. Kotera E, Tasaka M, Shikanai T (2005) A pentatricopeptide repeat protein is essential for RNA editing in chloroplasts. Nature 433: 326-330.
38. Tang J, Kobayashi K, Suzuki M, Matsumoto S, Muranaka T (2010) The mitochondrial PPR protein LOVASTATIN INSENSITIVE 1 plays regulatory roles in cytosolic and plastidial isoprenoid biosynthesis through RNA editing. Plant J 61: 456-466.
39. Cai W, Ji D, Peng L, Guo J, Ma J, et al. (2009) LPA66 is required for editing psbF chloroplast transcripts in Arabidopsis. Plant Physiol 150: 1260-1271.
40. Zehrmann A, Verbitskiy D, van der Merwe JA, Brennicke A, Takenaka M (2009) A DYW domain-containing pentatricopeptide repeat protein is required for RNA editing at multiple sites in mitochondria of Arabidopsis thaliana. Plant Cell 21: 558-567.
41. Takenaka M (2009) MEF9, an E-subclass pentatricopeptide repeat protein, is required for an RNA editing event in the nad7 transcript in mitochondria of Arabidopsis. Plant Physiol 152: 939-947.
42. Verbitskiy D, Härtel B, Zehrmann A, Brennicke A, Takenaka M (2011) The DYW-E-PPR protein MEF14 is required for RNA editing at site matR-1895 in mitochondria of Arabidopsis thaliana. FEBS Lett 585: 700-704.
43. Takenaka M, Verbitskiy D, Zehrmann A, Brennicke A (2010) Reverse genetic screening identifies five E-class PPR proteins involved in RNA editing in mitochondria of Arabidopsis thaliana. J Biol Chem 285: 27122-27129.
44. Hammani K, Colas des Francs-Small C, Takenaka M, Tanz SK, Okuda K, et al. (2011) The pentatricopeptide repeat protein OTP87 is essential for RNA editing of nad7 and atp1 transcripts in Arabidopsis mitochondria. J Biol Chem 286: 21361-21371.
45. Sung T-Y, Tseng C-C, Hsieh M-H (2010) The SLO1 PPR protein is required for RNA editing at multiple sites with similar upstream sequences in Arabidopsis mitochondria. Plant J 63: 499-511.
46. Zhou W, Cheng Y, Yap A, Chateigner-Boutin A-L, Delannoy E, et al. (2009) The Arabidopsis gene YS1 encoding a DYW protein is required for editing of rpoB transcripts and the rapid development of chloroplasts during early growth. Plant J 58: 82-96.
