# Supplementary material for: Elucidation of the RNA Recognition Code for Pentatricopeptide Repeat Proteins Involved in Organelle RNA Editing in Plants
Source: PLoS One. 2013 Mar 5;8(3):e57286. doi: 10.1371/journal.pone.0057286 (PMC3589468; doi:10.1371/journal.pone.0057286)
Supplement: Table S2 — List of Physcomitrella patens PPR proteins and newly characterized Arabidopsis PPR proteins, and their target sites. (PDF) [file pone.0057286.s008.pdf]

**Table S2.** List of PPR proteins and their target sites

| Name | Species* | AGI number | Localization | NSRs in the motifs | Target RNA sequence | Editing site | (ref) |
|---|---|---|---|---|---|---|---|
| PpPPR_56 | Pp | – | Mt | FVN-HNS-ANN-GMD-RTD-ITN-IVD-RTD-VND-ILD-RTN-VND-VVD-SND | uuuggaagucaccu<br>uauagacgguaucu | nad3eU230S<br>nad4eU272S | [23,26] |
| PpPPR_71 | Pp | – | Mt | RVN-YNT-VNS-ILD-RTD-SNN-ILD-RTD-VTD-IMD-LTD-VSD-VIH-PND-INN-VVT-KGD | uuccacaggugcuccuu | ccmFCeU122SF | [24] |
| PpPPR_77 | Pp | – | Mt | AVD-FND-INN-IID-RND-VND-VID-RTD-VNN-TLD-QND-ING-VLN-HND-IND-IAD-NND-MTV-SSD-GND-VNN-FVD-RAN-VND-STT-EGN | caauuacuaucaaagcuauuggacau<br>acuuuggcuuugaagcagcugcuugg | cox2eU370RW<br>cox3eU733RW | [23] |
| PpPPR_78 | Pp | – | Mt | SMD-YNT-HND-TMD-KNS-VTN-IID-ATD-INN-IVD-GND-ITN-VTD-HND-VND-VID-SNN-IND-VVT-EGN | uccugaggucuauauucuaa<br>acguuaugaauauuuuuuaa | cox1eU755SL<br>rps14eU137SL | [25] |
| PpPPR_79 | Pp | – | Mt | YVN-YNS-VND-FVD-TND-VTS-IMD-RTD-IND-ATD-RND-VTN-IMD-AND-VNN-AVT-KAN | acuuuucgacuauuuuu | nad5eU598RC | [25] |
| PpPPR_91 | Pp | – | Mt | SGN-NGD-YND-VSN-ILD-ATN-VTN-VVD-INS-MGN-VMD-STN-VTN-VND-VID-VTD-VNN-ITR-QGD | ugcacaaauaggauugcau | nad5eU730RW | [23] |
| MEF3 | At | At1g06140 | Mt | VSN-HND-FVD-YPN-VGD-LIS-YAN-VTN-CAD-VTN-ISN-VVE-EAM | aguucgaagaaga | atp4_89 | [47] |
| MEF7 | At | At5g09950 | Mt | YNN-VAN-YVD-VNN-VNT-YGD-FSN-LGS-EVM-GND-VNG-FIN-SNW-SGN-ISE-TND-VND-FAD-VSN-YND-VVR-ESN | ccuucauauuuuucguggucua<br>gagacgacuuuuucuugaacua<br>gcaaugacuuccuuuuucauua<br>uauuucacauuuucuaugauua | cytb_325<br>ccb206_28<br>nad2_1433<br>nad4L41 | [48] |
| MEF29 | At | At4g30700 | Mt | STD-FNN-SAE-LSD-IND-TLH-YTD-VNR-SVH-STS-PNN-VTS-YTN-VNT-VLS-KAG | uuugcugcuccaacca<br>gguguagccgcaauag | nad5_1550<br>cytb_908 | [49] |
| SLG1 | At | At5g08490 | Mt | RLC-EKW-ITS-VAD-LND-VNN-AAH-FNW-VAD-VID-SNW-ADD-VLE-KNT-VND-TSN-VMD-RGD-VTD-VTT-EAN | accuuuuucuuuccuugggca | nad3_250 | [50] |
| AHG11 | At | At2g44880 | Mt | FND-FTD-YTS-VTD-VNT-ITN-VND-VLK-KTQ-AND-ILK-EGN | This study | This study | |

*At, Arabidopsis thaliana; Pp, Physcomitrella patens.

**References**


23. Ohtani S, Ichinose M, Tasaki E, Aoki Y, Komura Y, et al. (2010) Targeted gene disruption identifies three PPR-DYW proteins involved in RNA editing for five editing sites of the moss mitochondrial transcripts. Plant Cell Physiol 51: 1942-1949.
24. Tasaki E, Hattori M, Sugita M (2010) The moss pentatricopeptide repeat protein with a DYW domain is responsible for RNA editing of mitochondrial ccmFc transcript. Plant J 62: 560-570.
25. Uchida M, Ohtani S, Ichinose M, Sugita C, Sugita M (2011) The PPR-DYW proteins are required for RNA editing of rps14, cox1 and nad5 transcripts in Physcomitrella patens mitochondria. FEBS Lett 585: 2367-2371.
26. Rudinger M, Szovenyi P, Rensing SA, Knoop V (2011) Assigning DYW-type PPR proteins to RNA editing sites in the funariid mosses Physcomitrella patens and Funaria hygrometrica. Plant J 67: 370-380.
47. Verbitskiy D, Merwe JA, Zehrmann A, Hartel B, Takenaka M (2012) The E-class PPR protein MEF3 of Arabidopsis thaliana can also function in mitochondrial RNA editing with an additional DYW domain. Plant Cell Physiol 53: 358-367.
48. Zehrmann A, van der Merwe J, Verbitskiy D, Hartel B, Brennicke A, et al. (2012) The DYW-class PPR protein MEF7 is required for RNA editing at four sites in mitochondria of Arabidopsis thaliana. RNA biol 9: 155-161.
49. Sosso D, Mbelo S, Vernoud V, Gendrot G, Dedieu A, et al. (2012) PPR2263, a DYW-Subgroup Pentatricopeptide repeat protein, is required for mitochondrial nad5 and cob transcript editing, mitochondrion biogenesis, and maize growth. Plant Cell 24: 676-691.
50. Yuan H, Liu D (2012) Functional disruption of the pentatricopeptide protein SLG1 affects mitochondrial RNA editing, plant development, and responses to abiotic stresses in Arabidopsis. Plant J 70: 432-444.
